# Recent Developments in Electrochemical Sensors for the Detection of Antibiotic-Resistant Bacteria

**DOI:** 10.3390/ph15121488

**Published:** 2022-11-29

**Authors:** Sekar Madhu, Sriramprabha Ramasamy, Jungil Choi

**Affiliations:** Department of Mechanical Engineering, Ajou University, Suwon 16499, Republic of Korea

**Keywords:** nanomaterials, electrochemical sensor, antibiotic-resistant, bacteria, antibiotic susceptibility testing

## Abstract

The development of efficient point-of-care (POC) diagnostic tools for detecting infectious diseases caused by destructive pathogens plays an important role in clinical and environmental monitoring. Nevertheless, evolving complex and inconsistent antibiotic-resistant species mire their drug efficacy. In this regard, substantial effort has been expended to develop electrochemical sensors, which have gained significant interest for advancing POC testing with rapid and accurate detection of resistant bacteria at a low cost compared to conventional phenotype methods. This review concentrates on the recent developments in electrochemical sensing techniques that have been applied to assess the diverse latent antibiotic resistances of pathogenic bacteria. It deliberates the prominence of biorecognition probes and tailor-made nanomaterials used in electrochemical antibiotic susceptibility testing (AST). In addition, the bimodal functional efficacy of nanomaterials that can serve as potential transducer electrodes and the antimicrobial agent was investigated to meet the current requirements in designing sensor module development. In the final section, we discuss the challenges with contemporary AST sensor techniques and extend the key ideas to meet the demands of the next POC electrochemical sensors and antibiotic design modules in the healthcare sector.

## 1. Introduction

**Figure 1:**
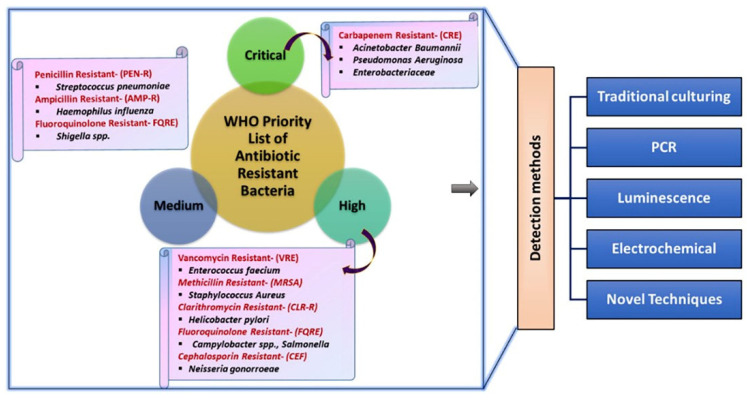
Potential antibiotic resistant bacteria and detection methods.

In the modern scenario, the healthcare, food production, and life expectancy of humans have become vulnerable due to the progressive intimidation of antimicrobial resistance (AMR). AMR is one of the foremost global health catastrophes, in which microorganisms can overcome antibiotic drug action against fatal infections [1,2]. The resistance mechanisms of these bacteria have evolved rapidly owing to selective pressure. Antibiotic defense mechanisms include the production of enzymes that deactivate antibiotics, such as various classes of β-lactamases or aminoglycoside-modifying enzymes, changes in antibiotic targets, and the reduction of intracellular antibiotic concentrations, either by limiting antibiotic entry or facilitating their excretion. Consequently, antibiotic-resistant microorganisms can survive and thrive [3,4]. Therefore, AMR infection causes serious illnesses and prolonged hospitalization, as well as increases the expenses of healthcare and second-line drugs, often leading to treatment failure. It is important for clinicians to understand the resistance mechanisms of these pathogens to choose appropriate antibiotic treatment, especially when the pathogen is known, but the antibiogram is still pending [5]. In the broad-spectrum, more than 1 million people, including 40% of infants, die annually due to AMR, and its associated global costs are anticipated to reach USD 100 trillion by 2050 [3,6]. The WHO recently released a list of potential globally resistant bacteria that require immediate advancement in drug design to address the growing global resistance to antimicrobial medicine (Figure 1) [7,8].

It also draws attention to highly perilous gram-negative bacteria, including *Acinetobacter*, the *Enterobacteriaceae* family, and *Carbapenemases*, as they are resistant to numerous conventional antibiotics compared to other dangerous gram-positive bacteria [9,10]. Generally, individuals infected with these resistant pathogens do not have any symptoms at the initial stage. Consequently, we found it difficult to stop the outbreaks before isolating the infected individual. Numerous clinical diagnostic and therapeutic methods have been used to identify the prevailing and evolving resistance traits and to adopt potentially life-saving antibiotic therapy [11]. The resistance of a pathogenic species to specific drugs was assessed using AST measurements. The current gold standards for AST are based on culture-based methods such as broth and agar dilution, rapid β-lactamase disk, and MIC gradient diffusion strip tests, which are generally based on monitoring the growth of bacteria that have formed either inhibition halos in agar plates or turbidity in liquid media, which are widely used [12,13,14,15,16,17]. Pure isolates should be used for culture enrichment, which requires several days. After inoculation of isolates onto agar or broth media, results were obtained in approximately 1–2 days [18,19,20]. However, in most instances, the tools employed are inadequate with protracted and expensive protocols and produce inaccurate results. Hence, preventive action, along with sensible antimicrobial susceptibility testing (AST) procedures, and the careful monitoring of these severe health threats is crucial, which requires continuous research and the discovery of new antibiotics to meet public health needs [11].

To address these issues and improve the standard of diagnostic-tailed restorations, several nonconventional methods have been developed. These assertions typically downplay the necessity of time-consuming measures such as enriching cultures and isolating pure cultures. The majority of these techniques use polymicrobial clinical samples that have not been purified and operate on nucleic acid hybridization or immunodiagnostic tenets [11]. AMR can be discovered in addition to an estimation of growth inhibition for the tested resistance to antibiotics from a brief cultivation procedure with predetermined antibiotic loading. Only end-point analysis is performed by the most rapid growth-based AST techniques, whereas others depend on routine sampling from the growing chamber. However, certain immunodiagnostic devices offer genuine online growth monitoring [21,22]. Recently, biosensor technologists have been working to provide a clear image of microbial metabolism, focusing on motility to offer a means of detecting alterations caused by locomotion and thermal stressors. Therefore, AST systems that are quick, dependable, simple to use, and affordable remain elusive.

To reduce barriers to accessibility, researchers are driven to create alternative tactics that are technically advanced, commercially viable, effective, accurate, and cost-effective. Among the non-conventional approaches, namely genomic sequencing, DNA amplification and hybridization technologies, microfluidics, and lab-on-chip techniques, electrochemical biosensor techniques have received considerable attention in AST [23,24]. It is advantageous owing to its sensitive and quick response, low cost with simple operational routes, and real-time data read to overlay the way for developing point-of-care testing (POCT) for distinctive and effective antibiotic drugs [25,26,27,28,29,30,31].

In addition, remarkable advances in novel materials, sensor platforms, and new technologies have made electrochemical sensors a valuable and powerful analytical tool. Nanostructured materials that enable electrochemical sensors have lower overpotential, faster electron transfer kinetics, and diffusion/mass transfer of the analyte compared to conventional electrochemical sensors, facilitating the development of sensitive and specific biosensors and novel sensing strategies [32,33,34]. Additionally, nanomaterials improve the stability, sensitivity, and selectivity of sensors in the presence of common interfering factors during electrochemical sensor development [35]. Advancements in materials science, and the subsequent availability of a wide variety of nanomaterials and composite materials with good electrical conductivity and/or catalytic activity, are primarily responsible for the improvements reported in detection limits as well as the specificity of electrochemical biosensors according to technological needs [36].

Since most reviews have elaborated on the significance of electrochemical and electrical sensors for pathogen detection, this review is focused on current electrochemical-based methods to detect antibiotic-resistant bacteria using various recognition probe elements in detail. In addition, we systematically reviewed the role of functional nanomaterials in effective resistant bacterial sensing and their therapeutic efficacy. Moreover, the bimodal ability of the nanoparticles and the novel strategies adopted to enhance the overall sensing performances of various resistant bacterial strains have been discussed. In conclusion, we summarized the prospects, difficulties in the present scenario, and new directions for future development in POCT.

## 2. The Basic Principle of Electrochemical Sensors for The Detection of Antibiotic-Resistant Bacteria

Electrochemical sensors are appealing for many applications where sensitivity, ease of operation, rapid response time, and low cost are crucial owing to the possibility of miniaturization and multiplexing, as well as the ability to construct flexible, disposable, and inexpensive electrochemical sensing devices. An electrochemical sensor is a device that can qualitatively or quantitatively analyze a target substance. It is essentially the sensing signal generated by the reaction of the measured substance with a specific sensing element, which is converted into an identifiable electrical signal proportional to the concentration of the target substance through a specific transducer. The complete electrochemical analysis system includes electrochemical sensing equipment, electrochemical detection instruments, and electrolytes. The electrochemical detection instrument, namely the electrode device, usually has a three-electrode structure, including a working electrode (WE), reference electrode (RE), and counter electrode (CE) [37].

**Figure 2:**
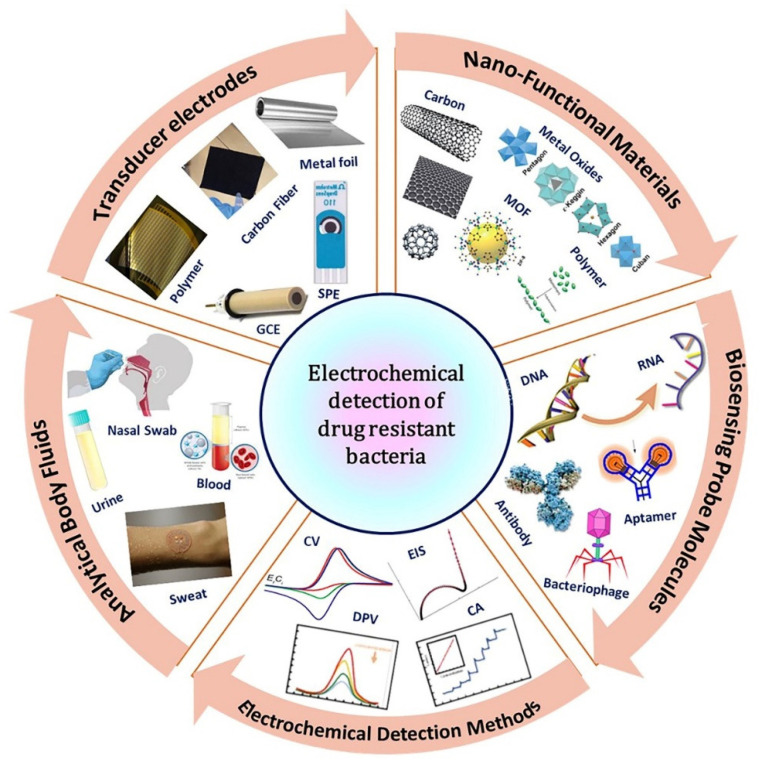
Electrochemical detection of drug-resistant bacteria.

The major classification of biosensors is based on receptor or transducer mechanisms (Figure 2). Based on receptors, biosensors can be classified into antibody, DNA, enzyme, whole-cell, and phage biosensors, whereas based on transducers, they can be classified into electrochemical, piezoelectric, calorimetric, and optical biosensors. Electroanalytical methods are the most important branch of analytical chemistry as it determines the characteristics as well as the amount of specific analyte(s) present in an electrochemical cell. The measurement of electrochemical features at the electrode interface reflects the relationship between the magnitude of the measured property and the concentration of specific chemical species. Based on the measurable signal, electrochemical detection of resistant bacteria is categorized as chronoamperometry, voltammetry, and electrochemical impedance spectroscopy (EIS) [38]. The amperometry (I-t) method is used to frequently detect resistant bacteria; a test substance undergoes an oxidation–reduction reaction at a constant potential and records the change in current with time [39]. Moreover, the commonly used voltammetry techniques in the detection of resistant bacteria [40,41] are: (i) Cyclic voltammetry (CV), which can simultaneously determine the redox peak potential and current and helps study the reversible redox reaction. (ii) Linear sweep voltammetry (LSV), a method of applying a linearly varied voltage to the electrode. (iii) Differential pulse voltammetry (DPV), which can provide relevant analyte information on the chemical form with high sensitivity. (iv) Square wave voltammetry (SWV), which applies a fast-scanning stepped voltage to the electrode and is more sensitive than CV. EIS is also an effective method for detecting resistant bacteria and can be used to analyze electrode performance [42]. Furthermore, an emerging analytical approach, photoelectrochemical bioassay, has also attracted considerable attention because of the difference between excitation and detection signals, remarkable sensitivity, and inherent miniaturization. In this technique, a semiconductor is excited by light to promote a catalytic oxidation reaction. In addition, the bacterial activities including growth and death can be monitored in real-time by measuring the capacitance changes.

To stay ahead of AMR challenges, contemporary mainstream antibiotic therapeutic strategies are responsible for their own regression by actively selecting resistant strains. This drives the need to support the continuous discovery of new antibiotics. In order to prolong the lifecycle of existing antibiotics, it is imperative that the research and development of new-generation antibiotics continues. Additionally, to reduce the existing demand for the development of new antibiotics, it is vital to implement effective control systems for antibiotic use [7]. In the following section, we discuss the different bio-probes used in the electrochemical detection of antibiotic-resistant bacteria.

### 2.1. Recognition Elements

Bio-probes are often referred to as the most crucial part of any biosensor as they determine the specificity of recognition for pathogen detection. High stability, simple immobilization on sensor platforms, and host-specificity with minimal cross-reactivity from interfering pathogens are ideal characteristics for any recognition element. Nucleic acids, antibodies, aptamers, and bacteriophages are the most widely used bio-probes for detecting resistant pathogens on biosensor surfaces [43].

### 2.2. DNA-Based Electrochemical Sensors

A DNA electrochemical sensor is an integrated receptor transducer that uses DNA as a biomolecule identifier to measure specific binding processes to target DNA by transmitting electrical signals [44,45]. The performance of a DNA-based biosensor is principally based on the immobilization of probe DNA to receptors that are oriented so that they are readily hybridized with its complementary target DNA. Accomplishing precise target recognition is a crucial step in DNA-based electrochemical sensor construction, which relies on specific immobilization strategies to ensure the optimal orientation of a probe DNA attachment on the working electrode surface [46]. Several DNA probe immobilization techniques have been employed in electrochemical DNA sensing, which can promote good reactivity such as adsorption methods, covalent bonding, and avidin–biotin interaction [47].

### 2.3. Antibody-Based Electrochemical Sensors

Antibodies are affinity biological recognition elements that have been used for a range of applications for more than two decades, owing to their strong antigen–antibody interactions [48]. Antibodies have the structure of immunoglobulins (Igs) in the form of a “Y” consisting of two heavy and two light polypeptide chains linked by disulfide bonds. Five classes of antibodies were defined and distinguished by their heavy chains: IgG, IgM, IgA, IgD, and IgE [49]. Among immunoglobulins, IgG, which is mostly used in developed immunosensors, has “Y”-shaped molecules in which two identical light chains and two heavy chains are linked by disulfide bonds as well as non-covalent interactions. Biosensors that have an embedded antibody as ligands or function in the antibody–antigen interaction are called immunosensors. Immunosensors are classified as label-free or labeled assays based on detection approaches. In label-free assays, the presence of an analyte is measured directly via biochemical reactions on a transducer surface, whereas in labeled assays, the analyte is trapped between the capture agent and the labeled agent with a special label such as an enzyme or nanomaterials to obtain a signal [50,51]. Antibody-based electrochemical immunosensors are typically made by immobilizing a recognition element (i.e., an antibody or antigen) on the surface of the working electrode based on the measurement of current and/or voltage resulting from the binding between the antibody and antigen [52,53,54,55].

### 2.4. Aptamer-Based Electrochemical Sensors

Aptamers are single-stranded DNA or RNA oligonucleotides that can bind specifically to a broad range of targets such as nucleic acids, proteins, metal ions, and other small molecules with high affinity, selectivity, and sensitivity [56,57]. Owing to these advantages over antibodies, aptamers are promising alternatives for most applications. They are structurally and functionally stable over a wide range of temperatures and storage conditions owing to their nucleic acid nature, which can be folded into two-dimensional (2D) and three-dimensional (3D) structures. A further feature of aptamers is that, depending on the detection criteria of the target molecule, they can be chemically modified [49]. Most aptamers are obtained through a combinatorial selection process called the systematic evolution of ligands by exponential enrichment (SELEX). SELEX has resulted in many aptamers that can bind to a wide range of targets, including metal ions, small molecules, peptides, proteins, and even complex targets such as whole cells and material surfaces [58,59].

Unlike antibodies, nucleic acid-based aptamers can withstand harsh operational conditions due to the comparatively rigid backbones and limited flexibility of nucleic acids compared to proteins, which have more torsional freedom and multiple conformational states concerning their backbones and side chains [60]. The outstanding molecular recognition ability of aptamers is due to their ability to adopt specific and complex three-dimensional shapes characterized as stems, loops, bulges, hairpins, pseudoknots, triplexes, or quadruplexes. These three-dimensional configurations allow the binding of targets ranging from small to large molecules such as peptides. Another advantage of nucleic acid aptamers is their excellent affinity for their targets, typically with dissociation constants ranging from picomolar to millimolar concentrations [61]. Aptamers are attractive for the development of biosensors because of their small size, high stability (especially DNA aptamers), high binding affinity and specificity, and easy modification.

### 2.5. Bacteriophages-Based Electrochemical Sensors

Bacteriophages are bacterial viruses that consist of single- or double-stranded DNA or RNA protected by a protein capsid that protects nucleic acids from the environment [62]. With a total population of approximately 10^31–33^, it is the most abundant and diverse entity in the biosphere. Apart from their abundance, their ability to survive adverse environmental conditions and multiply rapidly in their specific target host makes them ideal regulators of the microbial balance on Earth and indicators of the dynamic equilibrium between bacterial species [63]. Phages offer many advantages for biosensing, such as specificity in binding to their target host cells, ability to lyse and kill their hosts, and efficiency to replicate during infection. This makes phages valuable tools for the detection and identification of bacterial pathogens [64,65].

The aforementioned various probe molecules are incorporated with functional nanomaterials using novel strategies to develop the electrochemical sensor modules for the various resistant bacteria detections. In the following section, we will elaborate the significant properties of nanofunctional materials and sensing strategies in distinct pathogen detection.

## 3. Electrochemical Sensors for Resistant Bacterial Detection

### 3.1. Nanofunctional Materials-Based Sensor Platform

To circumvent the limitations of the gold-standard conventional systems, rapid identification and characterization tools are used in the screening of life-threatening microorganisms for effective treatment regimens. With the development and spread of emerging diagnostic systems for resistant infectious pathogens, effective POCT methods rely on robust optical and electrochemical sensing platforms [8,26,27,31,36,66,67]. Solicitation of functional nanomaterials in progressive biosensor technology signifies potential diagnostic methods in self-monitoring systems, which can enhance antibiotic usage and reduce patient demand for antibiotic prescriptions [68]. Interactions between nanotechnology and microbes have opened new possibilities for combating human diseases. In particular, modern nanomaterial-based AST approaches consider rapid and sensitive analysis outputs at affordable costs in disease management [8].

Nanomaterials and nanotechnology have evolved over the years and remain indispensable technology for major advances in science and technology. Nanomaterials are basic materials with one of their dimensions less than or equal to 100 nanometers. Nanomaterials have gained attention in technological advancements due to their tunable physicochemical properties including surface reactivity, electrical and thermal conductivity, quantum confinement effects, catalytic activity, mechanical stability, and biocompatibility in electrochemical sensor applications. Further, the notable physicochemical and functional properties of the distinct nanomaterials can be tuned using advanced synthetic approaches that are employed in various sensor applications. In addition, the materials can be functionalized and thus offer a great opportunity for combining biological recognition events and signal transduction mechanisms in the development of novel bioelectronic devices with excellent sensing properties [69,70]. The large surface-to-volume ratio of nanomaterials enables enhanced catalytic and sensing responses through the rapid movement of analytes through nanomaterial-based electrodes or sensors. In particular, the catalytic property of nanomaterials, the geometry, composition, oxidation state, and chemical/physical environment may play crucial roles in determining the catalytic activity and reactivity of nanomaterials, while the particle size and shape are also important considerations. Therefore, the relationship between these parameters and the catalytic performance of the nanomaterials may be -system dependent. In addition, a systematic understanding of the factor that controls catalyst reactivity and selectivity is essential. A key objective for the development of sensors with high sensitivity and selectivity is to modify the surface of the sensor electrode with functional nanomaterials, which involves amplifying the related electrochemical signal [71,72].

Recently, nanomaterials have been used in numerous clinical and POCT devices to increase the effectiveness of pathogen detection. In this regard, numerous nanomaterials including metal/metal oxides, carbon nanomaterials, dendrimers, magnetic nanoparticles, and polymer-based nanohybrid materials are employed along with diverse bioreceptors to modify the sensor electrode [73,74,75,76,77,78]. An effective interaction mechanism between the surface-modified electrode with these responsive probes and target molecules is imperative for the design and further improvement of the electrochemical performances. The following section provides an overview of the use of various functional nanomaterials and bioreceptors for the electrochemical detection of various antibiotic-resistant bacteria as well as a review of some of the major concepts that have been used to advance the perspectives of electrochemical sensors.

#### 3.1.1. AST-DNA Probe-Based Electrochemical Sensors

The rapid identification of drug-resistant bacteria can aid in the early diagnosis of several illnesses and offer a crucial direction for the effective administration of antibiotics. The genotypic or phenotypic modifications that occur in the bacterial system determine their resistance to antibiotic drugs, and the changes can be monitored through appropriate probes. Genotypic resistance arises in antibiotic-resistant bacteria owing to alterations in the nucleotides of the gene or mutations at the genomic level [7]. This direct approach of genotypic testing can be used to identify the resistance or susceptibility of bacteria; however, quantification of the resistance level is still inadequate. To overcome these limitations, several researchers have developed DNA probe-based electrochemical sensors to detect antibiotic resistance in bacterial pathogens in contrast to antibiotic drugs. In genotypic electrochemical sensors, the probe DNA is extracted from the intracellular genomic DNA as an immobilized sensor electrode, which can be used as a probe to bind with antibiotic-resistant genes or mutants. In electrochemical sensing, the target DNA hybridized with the probe DNA forms a duplex, which results in corresponding changes in impedance, current, or capacitance. These changes can be observed using different electrochemical sensing methods to quantify the target molecules. The precision in sensitivity, high reproducibility, prolonged shelf life, and applicability in real time are noteworthy aspects of electrochemical DNA detection [8]. An effective signal amplification strategy can be used to enhance sensitivity, which can be achieved via numerous tactics including tuning the functional properties of the sensor electrodes and the incorporation of enzymes or labeled dye molecules to enhance the catalytic reaction.

**Figure 3:**
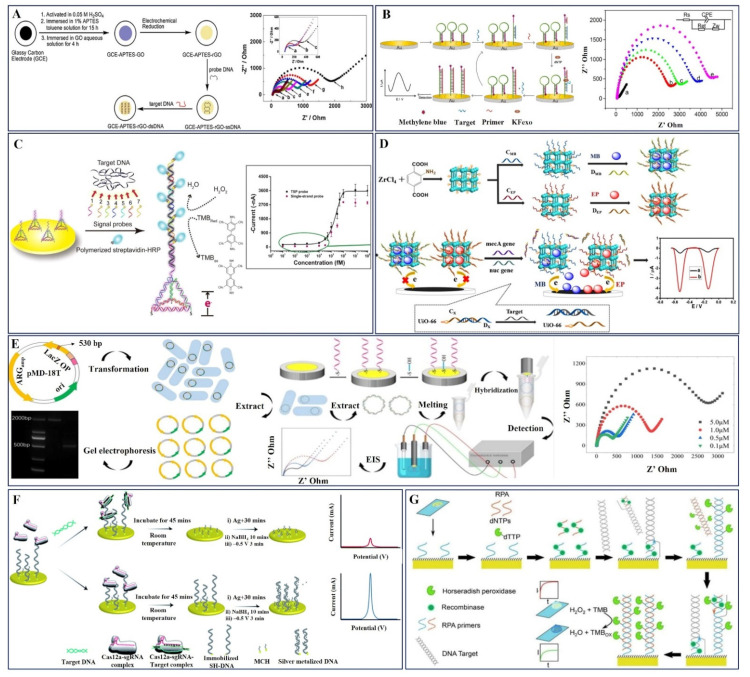
(**A**) Schematic illustration of the surface functionalization of the GCE for MRSA target DNA detection using the EIS technique. Copyright (2011) Elsevier [79]. (**B**) Graphical outline of the electrochemical *mecA* gene MRSA DNA biosensor fabrication process and EIS responses of surface-modified electrodes. Copyright (2015) Elsevier [80]. (**C**) Preparation of electrochemical DNA biosensor using a multi-signal probes (MSP) system containing 7 signal probes and a tetrahedral nanostructure-based capture probe for MRSA DNA detection using amperometric analysis. Copyright (2018) Elsevier [82]. (**D**) Synthesis of the DNA-gated UiO-66 and the schematic representation of simultaneous detection of *mecA* and *nuc* gene. MB methylene blue, EP epirubicin, CX (CEP, CMB) capture DNA, DX (DEP, DMB) displacement DNA. Copyright (2021) Springer [83]. (**E**) Preparation and extraction of ARGs and graphical representation of MCH-dsDNA-GE hybridized electrode construction and its EIS responses. Copyright (2022) American Chemical Society [84]. (**F**) Fabrication of amplification-free electrochemical CRISPR/Cas biosensor utilizing silver metallization (termed E-Si-CRISPR) to detect MRSA DNA. Copyright (2021) Royal Society of Chemistry [85]. (**G**) Illustration of modified recombinase polymerase amplification (RPA) reaction, coupled with amperometric electrochemical detection of the oxacillin resistance gene in *E. coli.* Copyright (2021) American Chemical Society [86].

Tuning the dimensions of nanomaterials plays a substantial role in improving their functional properties. Specifically, 1D and 2D nanomaterials exhibit notable changes in their physicochemical properties compared to their bulk form, which offers a preferable platform for bioreceptor immobilization [35,68]. Graphene is a unique 2D material that displays apparent physicochemical properties and serves as an essential sensor electrode for ample electrochemical pathogen detection applications. Predominantly, a reduced graphene oxide (rGO)-modified glassy carbon electrode has been reported by Zhijuan Wang et al. as a label-free detection platform for methicillin-resistant *Staphylococcus aureus* (MRSA) by conventional EIS (Figure 3A). The ssDNA genomic probe DNA was pre-adsorbed on the rGO with an APTES linker using a simple chemical method, which delivered high sensitivity and selectivity over a wide range with a limit of detection of 100 fM [79] (Table 1).

In another study, Ting Wand et al. proposed that the exceptional analytical performance of a distinctive isothermal strand-displacement polymerization reaction (ISDPR) approach captures the *mecA* gene in MRSA using E-DNA as a probe (Figure 3B). In addition, enhanced electrochemical sensitivity was achieved with the aid of self-assembled methylene blue (MB)-labeled hairpin probes on a gold electrode to enhance easy and fast electron transfer. Using the target recycling amplification strategy, the hairpin probes endured conformational changes during hybridization with the target DNA, resulting in a notable decrease in the electrochemical response. Primers located on the stem of the hairpin probes enabled the release of the target DNA to prompt the subsequent polymerization cycle. This target-recycling strategy in stimulated MB molecules produced an amplified current response corresponding to the *mecA* gene concentration [80]. An additional study was performed by Min Liu et al., in which they described the immobilization of *mecA* gene capture probes on the Au/GCE, followed by hybridization with MRSA target probes. Dual-labeled gold nanoparticles exhibited high-efficiency signal amplification to achieve outstanding selectivity and rapid sensitivity (less than 2 h) with a notable linearity of 50 pM to 250 pM, LOD = 23 pM, S/N = 3 [81].

Furthermore, a multi-signal probe (MSP) system comprising seven biotin-labeled signal probes was utilized by Li Zu et al. to develop a novel electrochemical sensor for the detection of mecA DNA in the MRSA genome. This MSP was pre-hybridized with the target DNA to form a complex, which was simultaneously attached to the electrode surface through a DNA tetrahedron structure probe (TSP) DNA (Figure 3C). The resultant electrocatalytic current response was measured with the assistance of streptavidin-labeled HRP enzyme and was found to be consistent with the target DNA concentration. The salient perception of MSP improved target accessibility in duplex DNA molecules, which delivered an amplified electrochemical signal. Here, the 3-D DNA TSP serves as the basis of the capture probe and offers constant support and optimal surface density for the MSP system and gDNA complex. This novel strategy can be used to frontier the usage of signal probes of at least three orders of magnitude with outstanding sensitivity, limit of detection (LOD-57 fM), and selectivity [82].

Enzyme-mediated amplification is a high-performance and cost-effective method for nucleic acid-based electrochemical sensing. Two-dimensional metal–organic frameworks are emerging nanozyme classes of materials with ordered chemical structures, adjustable sizes, and more active catalytic sites to effectively participate in sensor applications. Ge Dai et al. have used this enzyme—UiO-66-NH_2_ MOF—to fabricate a homogenous DNA sensor for MRSA detection (Figure 3D). Here, UiO-66-NH_2_ nano-carrier-encapsulated electroactive dyes, namely methylene blue (MB) and epirubicin (EP), amplify the electrochemical response, and N-doped porous carbon produced from Zn bimetallic zeolitic imidazolate frameworks is used to modify electrodes to increase electrocatalytic performance and sensitivity and achieve a higher recovery percentage in real sample analysis [83].

The self-assembled nanostructure on the electrochemical sensor electrode can provide larger active sites and ease the interaction of the analytes to strengthen the catalytic behavior of the sensor. A similar study was carried out by Chunli Wan et al., who reported the extraction of circular double-stranded β-lactum antibiotic-resistant genes (ARGs) from the universal primers using a PCR amplification method, which was self-assembled on a gold thin-film electrode for the detection of MRSA (Figure 3E). The proposed sensor demonstrated excellent detection ability with a good specific recognition ability for single-base, double-base, and three-base mismatched DNA [84]. Amplification-free and promising considerable ultrasensitive field-deployable electrochemical MRSA detection was demonstrated by Akkapol Suea-Ngam et al. using clustered regularly interspaced palindromic repeats (CRISPR) in addition to the silver metallization approach to developing the electrochemical sensor electrode (E-Si-CRISPR) (Figure 3F). Custom-made gRNA was used as a recognition probe for the detection of the MRSA target gene as shown in Figure 3F. Cas12a enzyme-mediated amplification provided superior analytical performance with linearity over five orders of magnitude (from 10 fM to 0.1 nM). Furthermore, this amplification-free sensor did not exhibit any degradation in the performance of real serum sample analysis [85].

A deleterious fragment of the oxacillin resistance gene was detected by Butterworth et al. using a modified recombinase polymerase amplification (RPA) reaction with horseradish peroxidase-labeled thymine nucleotides for DNA amplification (Figure 3G). It has been proven that an RPA reaction efficiently integrates nucleotides functionalized with large enzyme attachments and tolerates the presence of these bases. By producing double-stranded DNA at the electrode surface, this method eliminates the necessity of ssDNA production for post-amplification hybridization and minimizes the amount of end-user interaction. This solid-phase isothermal amplification reaction resulted in a remarkable limit of detection (319 CFUs/mL) with a minimum operation time of 60 min. Moreover, this rapid, easy-to-use technique can be implemented for screening clinical drug resistance genes in real time [86].

The electrochemical DNA sensing device demonstrated by Watanabe et al. is based on chronoamperometric (CA) detection of ferrocene-labeled probes coupled to gold nanoparticles (AuNPs). Sample DNA recovery in this DNA sensor system was made simple using magnetic nanoparticle (MNP)-modified probes. Using ferrocene-labeled AuNPs as probes, an electric signal may be generated, and MNP/DNA/AuNP conjugates may be formed by hybridization. The MNP/DNA/AuNP hybridization complex was magnetically separated, and the AuNP-ferrocene complexes were detected by electrochemical current responses. Dye-linked L-proline dehydrogenase (L-pro DH) was employed to create a very sensitive instrument by magnifying the current responses following a catalytic reaction with L-proline. This sensing technology and enzyme-mediated DNA diagnostic method were able to measure the target DNA from MRSA over a range of 10–166 pM [87].

Thus, the researchers have implemented several signal amplification strategies including isothermal and recombinase polymerase reactions, multi-signal probes, enzyme and nanozyme-mediated amplification, and self-assembled nanostructures to enhance the sensor performances. Hence, the distinct DNA probe molecules with appropriate sensing approaches can be used for the quantitative and qualitative electrochemical detection of resistant bacterial strains.

#### 3.1.2. AST-Antibody Probe-Based Electrochemical Sensors

**Figure 4:**
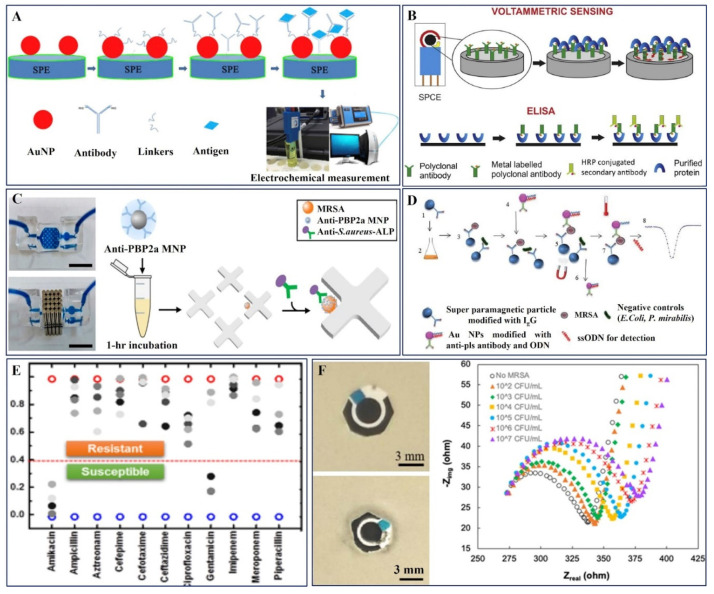
(**A**) Preparation of AuNP-modified carbon SPEs for the electrochemical detection of MRSA. Copyright (2020) Elsevier [88]. (**B**) Schematic representation of the electrochemical sensor using titania nanotubes modified SPCE coated with metal labeled antibodies-based MRSA detection. Copyright (2014) Elsevier [89]. (**C**) Photograph of bacterial capture device filled with dye in the absence and presence of an array of external magnets and anti-S. aureus antibodies functionalized with ALP integrated into the device. Copyright (2019) Elsevier [90]. (**D**) Scheme of MRSA detection using magnetic separation and electrochemical detection of oligonucleotides. Copyright (2016) Royal Society of Chemistry [91]. (**E**) Similarity measures estimated using the pattern matching algorithm for A. baumannii R4197. The dark color indicates a higher concentration. Blue and red circles indicate the similarity measure of cell-free media and A. baumannii R4197 without antibiotics. Copyright (2020) Springer, Nature Publishing Group [92]. (**F**) Immobilized phage SATA-8505 infectivity study and Nyquist plot for SATA-8505 modified electrode in the absence and presence of MRSA at different concentrations. Copyright (2021) The Electrochemical Society [93].

The growing need for reliable and fast AMR identification at POC systems depends on the use of antibody-based electrochemical sensors. The nanomaterial dimensions play an important role in tuning the performance of sensor devices. Khue et al. developed surface-modified screen-printed electrodes (SPEs) with Au nanoparticles (AuNPs) via a facile synthetic method followed by functionalization to immobilize the monoclonal MRSA-specific antibody (Figure 4A) for AST analysis. The electrochemical performance of the electrode revealed the prominence of the size of the AuNPS, which presented a linear detection range of 10–10^6^ CFU/mL, with an LOD of 13 CFU/mL [88].

Mandal et al. have established the metal oxide titania nanotubes (TiO_2_-NTs) for their generous improvement in the electrochemical detection of MRSA with the aid of a penicillin-binding protein, PBP2a, as shown in the schematic diagram, Figure 4B. The selectivity, sensitivity, and rapid detection capacity of the electrode were validated in the presence of diverse protein tyrosine phosphatases (PTP10D) and bovine serum albumin (BSA) [89].

The application of a microfluidic device and antibody-functionalized magnetic nanoparticles enabled the informal capture of MRSA, as presented in Figure 4C, which was developed by Nemr et al., and a strain-specific antibody functionalized with alkaline phosphatase for electrochemical detection was then used to identify MRSA. This method ensured that only bacteria belonging to the target strain and the resistance profile are measured. The approach has a turnaround time of less than 4.5 h, a limit of detection of 845 CFU/mL, and excellent discrimination against high quantities of common non-target nasal flora [90]. A sandwich immunoreaction of bacteria with IgG immobilized on streptavidin-coated polystyrene super-paramagnetic particles was prepared by Cihalova et al., and MRSA-specific antibodies were immobilized on gold non-magnetic nanoparticles labeled with oligonucleotides, which were detected by square wave voltammetry (SWV) in conjunction with the adsorptive transfer technique (Figure 4D). The antibody-based sensor device presented an early detection of MRSA at a concentration of 2 × 10^4^ CFU/mL, and the procedure can be adopted for the detection of any resistant bacteria [91].

Hence, the various nanostructured materials including metal oxides and magnetic nanoparticles have been effectively used as a transducer platform to identify the MRSA strains using the antibodies with enhanced sensitivity.

#### 3.1.3. AST-Aptamer Probe-Based Electrochemical Sensors

Lee et al. proposed an electrical AST (eAST) device through which 11 antibiotic-resistant bacteria were quickly screened for 6 h, as opposed to the extended (16 h) traditional AST procedures currently used in the field. This e-AST system created capacitance sensors using 60 functionalized aptamers as target-recognition probes. Clinical strains were obtained from septic patients using a pattern-matching algorithm and subsequent data validation using broth microdilution. A credible reference test was used to assess the performance of e-AST, and the results are shown in Figure 4E [92]. Consecutively, the functionalized aptamers-based capacitance sensors enabled the screening of eleven distinct antibiotic-resistant pathogens.

#### 3.1.4. AST-Bacteriophages Probe-Based Electrochemical Sensors

Bacteriophages are reliable diagnostic tools for AMR detection. A recent work published by Patel et al. described SATA-8505, a bacteriophage employed for the selective identification of prevalent surgical infection MRSA USA300 (Figure 4F) using a PEI-f-CNT sensor platform. The bacteriophage was immobilized on the working electrode by creating an electric field and by employing a charge-directed orientation technique. Infectivity studies using disk diffusion methods were performed to confirm the activity of the immobilized phage. There was a limit of detection of 1.23 × 10^2^ CFU/mL in aqueous solution and 1.29 × 10^2^ CFU/mL in blood plasma. For application in POC and other infections such as Pseudomonas aeruginosa, Klebsiella, and Listeria monocytogenes, the biosensing platform may be integrated into a lab-on-a-chip platform[93]. This sensor’s outcomes ensured the applicability of bacteriophages as novel sensor recognition probes in various resistant bacteria detection.

Several electrochemical sensor platforms have been established for the sensitive monitoring of potentially resistant bacterial strains. However, efforts have been made to develop an advanced target-resistant detection system that can kill bacteria, and the composition of another antibacterial unit is an effective strategy [94]. In this regard, nanomaterials are effective in identifying antibiotic-resistant bacteria and hold promise as possible antibiotics. The unique and versatile physicochemical characteristics of nanomaterials, including their size, shape, and surface chemistry, result in various bactericidal activities, making it harder for bacteria to resist treatments [95].

As a result, nanoparticles can contribute to therapy by increasing their interaction with the bacterial cell system, and the prospective candidates design the electrochemical sensor electrode, which may increase the effectiveness of the regimen.

Therefore, to develop potential nanomaterial-based antibiotics against resistant bacteria, it is essential to understand the cell wall properties of gram-negative and gram-positive bacterial strains. Typical studies have demonstrated that the main mechanism underlying the antimicrobial effects of various NPs is the generation of ROS, induction of cell membrane penetration, disruption of bacterial cell membranes, induction of intracellular antibacterial actions, and interactions between proteins and DNA [96]. In this context, researchers have focused on developing NPs that have been conjugated to the antibiotic with greater antibacterial activity compared to the primal antibiotic or NPs. This hints at a synergistic impact and suggests that antibiotics and NPs have separate antibacterial mechanisms.

### 3.2. Bimodal Action of Nanomaterials-Electrochemical Sensor and Antibiotics

**Figure 5:**
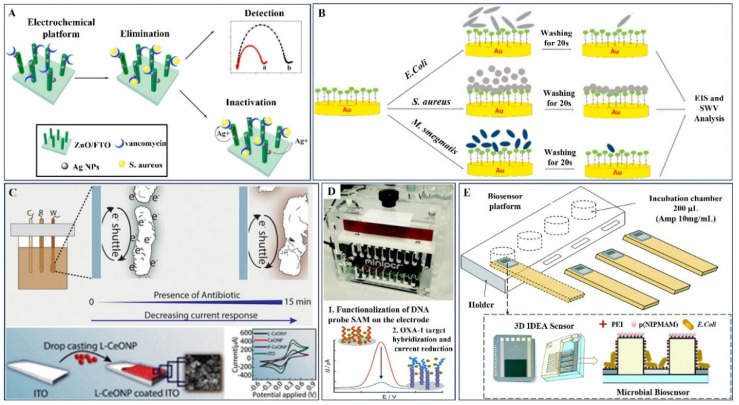
(**A**) Schematic description of the developed electrochemical sensor platform for simultaneous detection, elimination, and inactivation of *S. aureus*. Copyright (2017) Elsevier [97]. (**B**) Interaction between Screen Printed Gold Electrodes-*Van* with different bacteria. Copyright (2021) Elsevier [98]. (**C**) Stepwise fabrication of L-CeONP/ITO working electrode by CV method for electrochemical analysis of *E. coli* in presence of antibiotics. Copyright (2020) American Chemical Society [100]. (**D**) Mini PCR machine used for DNA amplification and portrayal of the DNA binding process and DNA measurement using the DPV technique. Copyright (2019) Royal Society of Chemistry [101]. (**E**) Fabrication of a microbial biosensors platform to monitor the bacterial response to ampicillin. Copyright (2019) Royal Society of Chemistry [102].

In view of this, Zhiqing Yang et al. have investigated the electrochemical sensing ability of 3D ZnO nanorod arrays (3D-ZnO) decorated with antimicrobial agent silver nanoparticles (AgNPs), functionalized with antibiotic drug vancomycin (Van) for treating bacterial infections including *Staphylococcus aureus* (SA) (Figure 5A). Owing to the composite antibacterial the AgNPs and Van units’ synergistic germicidal impact through bimodal, the platform has displayed significant antibacterial activity (99.99%). This could be possible through the bimodal synergetic effects of Ag NPs count on cell wall rupture followed by sequential penetration and ROS production, which kill the bacteria [96,97] (Table 2). Additionally, in a study by Dizaji et al. screen-printed gold electrodes were modified with thiolated Van molecules using the self-assembly monolayer technique (Figure 5B). Impedance analysis confirmed the proof of concept from the charge transfer ability towards the bacterial strains *Escherichia coli*, *Staphylococcus aureus*, and *Mycobacterium smegmatis* revealed the high AST efficiency of the electrode. The proposed work promises whole-cell detection of Van-susceptible bacteria in real-time [98,99].

A study by Rao et al. illustrated rapid AST detection within a 15 min assay time, namely EAST, which is used for live monitoring with the assistance of a time--lapse microscopy video (Figure 5C). Gram-positive *Bacillus subtilis* and Gram-negative *Escherichia coli* were used as model organisms in the suggested EAST with success in tracking bacterial concentration, decay kinetics in the presence of different antibiotics (ciprofloxacin, cefixime, and amoxicillin), medication efficacy, and IC_50_. The colony-counting technique was used to validate the kinetics of bacterial decomposition in the presence of antibiotics. As a working electrode, indium tin oxide (ITO) coated with bacteria-friendly L--lysine-functionalized cerium oxide (CeO) nanoparticles was used to investigate the improved electron transfer rate in EAST [100].

Butterworth et al. introduced SimpleStat, an open-source electrochemical platform with a highly simplified design that was programmed to perform differential pulse voltammetry (DPV) and was used to find OXA-1 DNA sequences for oxacillin resistance, as shown in Figure 5D. Polycrystalline gold electrodes and gold-plated PCB electrodes were integrated into a simplified SimpleStat printed circuit, which was used in further tests. The study revealed that in contrast to the *tetA* gene, which codes for tetracycline resistance, this DNA sensor can be utilized to specifically detect OXA-1 [101].

A novel label-free biosensing platform based on a microbial biosensor method was demonstrated by Brosel-Oliu et al. (Figure 5E) and evaluated by performing antibiotic detection bioassays in the diluted solutions. A 3D interdigitated electrode array (3D-IDEA) impedimetric transducer with immobilized *E. coli* bacteria served as the foundation of the microbial biosensor. The electrode digits in the 3D-IDEA are separated by insulating barriers to maximize the sensitivity to surface impedance changes. To concentrate bacteria, increase the reproducibility of *E. coli* immobilization, and improve the sensitivity for monitoring bacterial response, a unique technique was used to selectively immobilize bacteria in the gaps above the electrode digits between the barriers, referred to here as trenches. This work suggested that the initial anchoring layer of a highly absorbent material should be used in trenches for optimal bacterial attachment [102].

**Figure 6:**
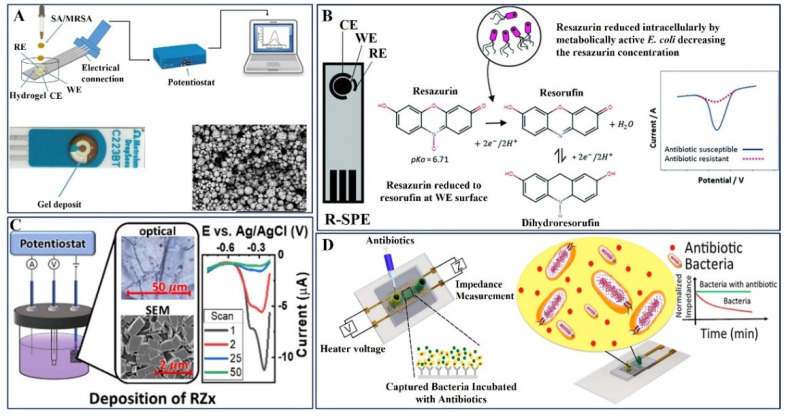
(**A**) Schematic depicts the bacteria pipetted onto the electrode, and electrochemical measurements were performed using a potentiostat, electrode surface modified with agarose gel deposit, and SEM image of bare Au DropSens electrode. Copyright (2019) Elsevier [103]. (**B**) An overview schematic of phenotypic AST using an electrochemical-based sensing methodology. Copyright (2021) Royal Society of Chemistry [104]. (**C**) Electrochemical deposition of RZx using sequential DPV method (Inset: Optical and SEM images of RZx/PGS) and corresponding DPV voltammogram. Copyright (2021) Elsevier [105]. (**D**) Antibiotic susceptibility measurement using label-free electrical sensing on a biosensor without surface modification and its impedance monitoring. Copyright (2017) American Chemical Society [106].

Hannah et al. reported a method to monitor bacterial growth and determine antibiotic susceptibility: drug-resistant *S. aureus* cultures were deposited onto agarose gel-modified electrodes containing therapeutically significant drugs (Figure 6A). According to these findings, *S. aureus* can grow on electrodes modified with gel that does not include any antibiotics but is inhibited when the antibiotic is seeded into the electrode modified with gel. In contrast, MRSA drug-resistant strains can grow on electrodes modified with gels that contain clinically significant levels of antibiotics. The results indicate that quick growth profiles with potential antibiotic susceptibility result in less than 45 min, which is a significant improvement over the current gold standards of at least 1–2 days [103].

Multidrug-resistant bacteria that causes urinary tract infections (UTIs) can be identified using current phenotypic approaches, which can take up to 48 h. The creation of resazurin bulk-modified screen-printed macro electrodes (R-SPEs) prepared by Crane et al. revealed an efficient electrochemical detection platform for the assessment of antibiotic susceptibility in complicated UTIs, according to the novel inquiry (Figure 6B). Resazurin was found using differential pulse voltammetry (DPV) down to 15.6 M. R-SPEs were used to perform antibiotic susceptibility testing (AST) on *E. coli* (ATCC^®^ 25922) using DPV to measure the relative amounts of Resazurin between bacteria that did and did not receive antibiotic treatment. After a total of 90 min, including the inoculation of artificial urine, preincubation, and testing period, antibiotic susceptibility was assessed using R-SPEs [104].

Existing efforts call for the addition of antibiotic-interfering redox-active compounds to the solution. Using pyrolytic graphite sheets (PGS), a simple electrodeposition method was adopted by Bolotsky et al. to produce a redox-active crystalline layer (designated as RZx), which was then used as the sensing layer for reagent-free electrochemical AST, as schematically represented in Figure 6C. The sensors with *E. coli* K-12 treated with two antibiotics, ampicillin and kanamycin, were tested to demonstrate proof-of-concept. While the pH-sensitivity of RZx (53 mV/pH) primarily allows the sensors to detect bacterial metabolism, secreted redox-active metabolites/compounds from entire cells may also contribute to the signal. The sensors provide a precise prediction of the minimum inhibitory concentration (MIC) in 60 min (p 0.03) by tracking DPV signals [105].

An advanced label-free biosensor was designed by Safavieh et al. for the rapid AST analysis of the bacteria isolated from whole blood, and the schematic illustration is given in Figure 6D. Using printed electrodes on flexible plastic microchips, the target bacteria were trapped using antibodies after 30 min, and their electrical response was observed in both the presence and absence of antibiotics over the course of an hour of incubation. *E. coli* and MRSA were used as clinical models for testing the microchip together with medications such as ampicillin, ciprofloxacin, erythromycin, daptomycin, gentamicin, and methicillin. The outcomes were evaluated in comparison with current best practices, such as testing for bacterial viability and traditional antibiograms. By identifying the appropriate antibiotics for infections, the method described here has the potential to deliver accurate and quick bacterial screening, as well as clinical therapy guidance [106].

Using solid-phase isothermal primer elongation with redox-labeled oligonucleotides, Ortiz et al. newly reported the electrochemical detection of single-point mutations linked to rifampicin resistance. Four 5′-thiolated primers were self-assembled by chemisorption on the gold electrodes of an array. These primers were created to complement the same fragment of the target sequence and were varied only in the final base, addressing the polymorphism site. Only at the electrode, where there was complete complementarity between the surface-tethered probe and the target DNA being interrogated, was the Klenow (exo-) DNA polymerase-mediated primer extension with ferrocene-labeled 2′-deoxyribonucleoside triphosphates (dNFcTPs) observed after hybridization with single-stranded DNA. After 20 min of hybridization, Klenow (exo-) DNA polymerase-mediated primer elongation at 37 °C for 5 min was ideal for the enzymatic incorporation of a ferrocene-labeled nucleotide, leading to unmistakable electrochemical detection of a single-point mutation in 14 samples of genomic DNA isolated from *Mycobacterium tuberculosis* strains. Multiplexed electrochemical single-point mutation genotyping can be performed using this method [107].

Here, Xin Li et al. created a bioassay based on a smart pH-regulated switchable photoelectrochemical (PEC) platform that offered ultrasensitive detection of two prevalent penicillin-resistant gene subtypes, *bla_CTX-M-1_* (target 1, labeled as T_DNA1_) and *bla_TEM_* (target 2, labeled as T_DNA2_). The pH-sensitive antimony tartrate (SbT) complex-grafted silica nanospheres (S_DNA1_-Sb_T_@ SiO_2_NSs) served as signal DNA1 tags in this bioassay. The switchable dissociation of the pH-responsive S_DNA1_-SbT@SiO_2_NSs complex under external pH stimuli is essential for PEC bioassay operations because it initiates the pH-regulated release of ions that have been inserted in sandwich-type DNA nanoassemblies. The release of embedded SbO^+^ was triggered by the dissociation of S_DNA1_ tags (ON state) under acidic conditions. The S_DNA1_ tags were prevented from dissociating under alkaline conditions (OFF state). Using the metal ion release caused by DNA hybridization, the target T_DNA2_ was found. Hg^2+^ is released when the inserted hairpin T-Hg^2+^-T fragment unwinds and fuses with the second anchored DNA signal (S_DNA2_). By exchanging ions with the photosensitive ZnS layer, the released SbO^+^ or Hg^2+^ ions would cause the formation of Sb_2_S_3_/ZnS or HgS/ZnS heterostructures, leading to amplified photocurrents and ultimately realizing the ultrasensitive detection of penicillin-resistant gene subtypes, bla_CTXM1_, and bla_TEM_. It shows significant promise for creating a new class of genetic POC devices by effectively measuring bla_CTXM1_ and bla_TEM_ in actual *E. coli* plasmids[108].

An electrical capacitance sensor was developed by Jo et al. and integrated with the aptamer probe that can track bacterial growth and antibiotic susceptibility. This rapid measurement of capacitance by the inhibition of *E. coli* and *S. aureus* bacterial growth enabled rapid AST monitoring in real time [109]. In a similar study, the graphene dispersion was mixed with *E. coli* cells deposited on GCE by Li et al. to identify resistant bacteria in the presence of common antibiotic drugs, namely ofloxacin, penicillin, and cefepime. Electrochemical reduction in the presence of antibiotics revealed the sensing ability of the graphene–*E. coli*/GCE platform [110].

Furthermore, Besant et al. provided a unique electrochemical method that allows for an hour-long fast readout of the antibiotic susceptibility profile of a bacterial infection. A redox-active substance was measured electrochemically to determine the concentration of metabolically active bacteria. Miniaturized wells are used to collect bacteria, which are then treated with antibiotics and checked for resistance. With clinically important numbers of bacteria, this electrochemical phenotyping method yields results that are comparable to those of culture-based analysis [111]. Recently, Mishra et al. measured the metabolic activity of live bacterial cells electrochemically by utilizing the electroactive redox dye resazurin and platinum (Pt) sputtered electrodes for the screening of two different bacterial strains of *Klebsiella pneumonia* (ATCC-700603) and *E. coli* (ATCC-25922) against the antibiotics, namely ampicillin, kanamycin, and tetracycline. The rapid electrochemical reduction current response provides high sensitivity and quick susceptibility monitoring compared to the time-consuming traditional disc diffusion method[112]. In another study, Ikeuchi et al. used Hoechst dye-impregnated SPCE to capture the MRSA genomic probe mecA using a hand-held potentiostat within two minutes. Another noteworthy point is that the sample volume for analysis is merely 10 µL, which can be obtained within an hour from nasal swab samples for the active surveillance of MRSA and Methicillin-resistant *Staphylococcus epidermidis* (MRSE) carriers [113].

Current research trends indicate an understanding of the salient features of nanomaterials, which are precisely tailored to enhance the therapeutic effect of the new class of antibiotics while minimizing toxicity to the host due to their tunable properties, especially their surface functions. Nanoparticles have access to antibiotic modalities that are unfamiliar to bacteria and are therefore not part of their standard arsenal of defense. Compared to conventional antibiotics, nanoparticles can avoid current resistance mechanisms and may be less likely to select for resistance. Accordingly, the combination of antimicrobial nanomaterials with various antibiotics in practice and the newly emerging photoelectrochemical approach can be used for the design of resistant bacteria sensors and effectively applied for bacterial growth and death monitoring in real-time.

**Table 2:** List of bimodal action of nanomaterials in electrochemical sensors and antibiotics.

S. No	Working Electrode	Antibiotic	Target Bacteria	Probe	Electro Chemical Method	Detection Range	LOD	Interference	Body Fluid	Ref.
1.	AgNPs/3D-ZnO Check	Vancomycin	*S. aureus*	*Van*	EIS	1000–2000 CFU/mL	330 CFU/mL	*E. coli*	--	[97]
2.	Thiolated vancomycin/SPGE	Vancomycin	*S. aureus*	*HS-Van*	EIS	10^1^ to 10^8^ CFU/mL	<39 CFU/mL	--	--	[98]
3.	L-lysine coated CeO/ITO (EAST)	Ciprofloxacin, Cefixime, Amoxycillin	*E. coli* and *B. Sutbilis*	--	CV	0.001×10^6^–10 ×10^6^ CFU/mL for *E. coli* and 250 ×10^12^–280 ×10^12^ CFU/mL for *B. Sutbilis*	--	*--*	--	[100]
4.	Polycrystalline gold electrode	Oxacillin	*OXA-1 DNA*	Complementary *OXA-1* DNA	DPV	--		DNA from the *TetA* gene	--	[101]
5.	*E. coli* Bacteria/PEI/p(NIPMAM/PDMS microgel (3D-IDEA)	Ampicillin	*E. coli*	PEI/p(NIPMAM/PDMS microgel	EIS	2–8 mg/L	2 mg/L	ampicillin resistant and non-resistant *E. coli*	--	[102]
6.	Agarose gel modified Au electrode	Amoxicillin, Oxacillin	*S. aureus*, MRSA	--	EIS and DPV	8 μg/mL and 50 μg/mL	--	--	--	[103]
7.	Resazurin-modified graphite SPE	Gentamicin	*E. coli*	Resazurin	DPV	0–1000 μM	15.6 μM	--	Artificial Urine	[104]
8.	Nafion coated RZx on graphite sheets	Ampicillin, Kanamycin	*E. coli*	--	DPV	0.001–10 μM	16 μg/mL	--	Whole blood, Milk	[105]
9.	Silver interdigitated carbon working electrode	Ampicillin, ciprofloxacin and Erythromycin, Daptomycin, Gentamicin, Methicillin	*E. coli* and MRSA	Label free	Normalized EIS	0.1 μM–100 μM	0.1 μM	single-base, double-base, and three-base mismatch DNA	Whole blood, Human urine	[106]
10.	Au electrode	Rifampicin	*Mycobacterium tuberculosis*	Solid-phase isothermal primer	SWV	6 μM–140 μM	6 μM	A mixture of the four dN^Fc^TP	--	[107]
11.	S_DNA1_-SbT@SiO_2_NSs complex and Sb_2_S_3_/ZnS/ITO	Penicillin	bla-_CTX-M-1_ and bla-_TEM_	DNA	Photo electrochemistry	1 nM to 10 μM	1 nM	Acidic pH for bla-_CTX-M-1_ and alkaline pH for bla-_TEM_	Plasmid	[108]
12.	Au electrode/glass substrate	Gentamicin	*E. coli*, *S. aureus*	Aptamer	Capacitance	0–50 μg/mL	-	Aptamer of *A. baumannii* and *E. faecalis*	--	[109]
13.	GCE	Ofloxacin, Penicillin, Cefepime	*E. coli*		DPV	1 × 10^5^ CFU–5 × 10^7^ CFU/mL	10 CFU/mL	--	--	[110]
14.	Miniature incubation chamber WE	Ampicillin, Ciprofloxacin	*E. coli*, *Klebsiella nueumoniae*		DPV	1–1000 CFU/mL	1 CFU/mL	--	Human Urine	[111]
15.	Pt/Ti/Glass	Ampicillin, Kanamycin, tetracycline	*E. coli*, *Klebsiella nueumoniae*		DPV	0–0.9 mM	0.12 mM	--	--	[112]
16.	SPE	Methicillin	*mecA* DNA from MRSA	*mec A1* and *mec A2* Primer	CV	3 × 10^4^–3 × 10^6^ CFU/mL	-	NTC negative control	Nasal swab	[113]

## 4. Summary and Future Outlook

The detection of pathogens is becoming more important in clinical research, food safety, and environmental monitoring. A major challenge in pathogen diagnostics is the rapid and accurate characterization of antibiotic resistance of infecting species or strains in different environments to prescribe appropriate antibiotics to patients and regions at the early stages of infection. Despite the cost, automation, and other advances in traditional culture methods, advanced microscopy-based techniques are used in practice for detecting pathogen resistance, and growth monitoring is still time-consuming and labor-intensive.

With such challenges and efforts to overcome these limitations, we believe that the electrochemical sensors will continue to evolve and facilitate enhanced AST device performances even in resource-limited regions.

In this review, we emphasize the importance of electrochemical techniques and the role of nanomaterials used for the sensing of various antibiotic-resistant bacterial strains. Although a direct device is yet to be developed to detect drug resistance, advancements in the last decade have brought us closer to understanding electroactive drug compounds and detecting drug resistance using electrochemistry.

We can infer from this review that the use of nanostructured materials in electrochemical sensors, as well as modifying their size, shape, and other functional aspects, can significantly improve system performance. Nanomaterials play a vital role in current sensor technologies and further meet the demands of developing transportable, fully automated, implantable, and/or wearable devices for efficient pathogen screening.

In this study, we discussed the significance of different accessible biorecognition probes employed in electrochemical-resistant bacteria sensing, in which numerous reports available on DNA-based systems highlighted the various effective approaches in material preparation and signal amplification to accomplish the improved sensitivity and selectivity. However, the applicability of these systems in real samples is still being investigated. In contrast, only a few reports are available on aptamer and bacteriophage-based systems and their effective resistant bacteria screening, where there is still room for implementing and enhancing the system performance. Sterile body fluids are susceptible to serious invasive bacterial infections and are critical, with high morbidity and sequelae risks. To the best of our knowledge, the effectiveness of the sensor in detecting bacteria in various clinical samples is still unclear. Therefore, efforts must be made to assess the efficacy of the present methods for detecting antibiotic-resistant bacteria in various clinical samples of human bodily fluids. Also, the use of electrochemical sensors further enables the development of multiplexed rapid POC devices to identify the presence of bacteria in clinical human body fluids.

In a nutshell, combining stimuli-responsive nanoparticles with traditional antibiotics in an efficient manner can provide a cutting-edge new class of antibiotics that will serve the present demand for medical treatments and monitoring systems. One of the biggest obstacles to its clinical use is nanomaterial-based antibiotic therapies, which severely compromise systemic safety through their possible accumulation and formation of bioconjugates that can affect the functions of human biological systems. Therefore, while developing novel therapies based on nanomaterials to combat antibiotic-resistant bacteria, researchers should perform cytotoxicity analysis and confirm the dosage optimization and clearance of nanoparticles from the biological system after intravenous administration.

## Figures and Tables

**Table 1:** Nanomaterial-based electrochemical sensors transducer electrode for the detection of antibiotic resistance bacteria.

S. No	Working Electrode	Antibiotic	Target Bacteria	Probe	Electro Chemical Method	Hybridization Time	Detection Range	LOD	Interference	Body Fluid	Ref.
1.	GCE-APTES-rGO-dsDNA	Methicillin	*DNA from MRSA S. aureus*	ssDNA	EIS	30 min	0.1 pM–1 μM	0.1 pM	n-DNA	--	[79]
2.	*mecA* gene/MCH/hairpin probe/Au electrode	Methicillin	*mecA DNA from MRSA*	E-DNA	SWV	2 h	0–400 pM	63 fM	one-base mismatched (T2), three-base mismatched (T3), and non-complementary(T4) DNAs	--	[80]
3.	*mecA* gene/Au/GCE	Methicillin	*mecA DNA from MRSA*	*mecA* gene	DPV	--	50–250 pM	23 pM	one-base mismatch and complementary DNAs	--	[81]
4.	MSP-TSP/Au electrode	Methicillin	*130 nt synthetic ssDNA* and *gDNA*	Multi-Signal Probes	EIS	4 h	100 nM–10 fM	10 fM and 57 fM	Non-complementary *E. coli* gDNA	--	[82]
5.	UiO-66/BMZIF-derived NPCs	Methicillin	*mecA* and *nuc* gene DNA from MRSA	*ss*DNA	DPV	1 h	5–1 × 10^5^ fM	1.6 fM and 3.6 fM	One (T1), and three bases (T2) mismatched and non-complementary DNA (T3)	--	[83]
6.	MCH-sDNA-GE	Ampicillin	*β-lactam* gene	*ss*DNA-GE	EIS	1 h	3.1–480 pM	3.1 pM	single, double, and three-base mismatch DNA	--	[84]
7.	E-Si-CRISPR	Methicillin	*mecA* DNA from MRSA	Aptamer gRNA	SWV	45 min	10 fM–0.1 nM	3.5 fM and 10 fM	Colonies of *E. coli*, *E. Faeclias*, *L. Monocytogens* and *S. epdermidis*, *AND MSSA*	Lysate and Human serum	[85]
8.	Screen printed Au SPGE	Oxacillin	*DNA* AMR gene sequence *E. coli*	Solid-phase RPA primers	Amperometry	12 h	319−20,830 CFU/mL	319 CFU/mL	--	--	[86]
9.	MNP/DNA1-Au/DNA-2	Methicillin	*mecA* DNA from MRSA	Ferrocene-labeled probes	CV	--	10–166 pM	10 pM	DNA from *S. aureeus* and *E. coli*	--	[87]
10.	Au/SPCE	Methicillin	Antigen	Monoclonal anti-MRSA antibody and Aptamer gRNA	CV, DPV	--	10–10^6^ CFU/mL	13 CFU/mL	*E. coli* O157:H7		[88]
11.	TiO_2_-NTs	Methicillin	*S. aureus*	PBP2a Protein	CV	--	1–100 ng/μL	1 ng/μL	Recombinant protein PTP10D	--	[89]
12.	Au electrode	Methicillin	PBP2a antibody	Monoclonal anti-MRSA antibody	DPV	--	3–10^5^ CFU/mL	3 CFU/mL	Nontarget strains MSSA, MSSE, and MRSE	Nasal swab	[90]
13.	Au nanoparticles modified by anti-Pls	Methicillin	Antigen	MRSA-specific antibody	SWV	--	0.2–10 μM 4 × 10^7^–2 × 10^4^ CFU/mL	2 × 10^4^ CFU/mL	*E. coli* and *P. mirabili* ODN	--	[91]
14.	e-AST system on Au	11 antibiotic drugs	*E. coli U433*	60 aptamers	Capacitance	--	0.5–128 mg/mL	--	*--*		[92]
15.	PEI-f-CNT	Methicillin	MRSA USA300 strain	*SATA-8505*, *bacteriophage*	EIS	--	10^2^–10^7^ CFU/mL	1.23 × 10^2^ CFU/mL in aqueous solution 1.29 × 10^2^ CFU/mL in blood plasma	SATA-8505’s nonhost organisms as *E. coli* and *P. putida*	Blood plasma	[93]

## Data Availability

Data availability not applicable.
